# Lessons learnt from advocating for family medicine in South Africa

**DOI:** 10.4102/phcfm.v17i1.4795

**Published:** 2025-01-17

**Authors:** Robert J. Mash, Klaus Von Pressentin, Jenny Nash, Tasleem Ras

**Affiliations:** 1 Division of Family Medicine and Primary Care, Faculty of Medicine and Health Sciences, Stellenbosch University, Stellenbosch, South Africa; 2 Division of Family Medicine, University of Cape Town, Cape Town, South Africa; 3 Amathole District Clinical Specialist Team, Department of Health, Eastern Cape, East London, South Africa

**Keywords:** family physicians, advocacy, government policy, human resources for health, district health services

## Abstract

South Africa has one of the most established family medicine disciplines in the region, with well over 1000 people on the register. Nevertheless, by international standards, the number of family physicians per 10 000 population is low and there is still a need to advocate for the contribution of family physicians to the health system. The speciality of family medicine was promulgated in 2007 after many years of advocacy. Advocacy has continued with a focus on human resources for health policy and deployment of family physicians in district health services. In the private sector, there is also advocacy for the scope of practice and proper remuneration of family physicians. This short report reflects on the lessons learnt in terms of seven key principles for advocacy with government: understand the issue, identify the right audience, build relationships, use evidence and data, craft a clear message, engage the public, and use the media.

## Introduction

South Africa has one of the largest groups of family physicians on the continent, with well over 1000 people on the Health Professions Council’s register.^1^ Nevertheless, the number of family physicians per 10 000 population is still low by international standards at 0.16/10 000 and they are unevenly distributed between provinces.^1^ South Africa recognised family medicine as a speciality in 2007 and this historic decision was preceded by many years of advocacy. Since then, roughly 20–30 new family physicians have been trained per year across nine training programmes.^1^ Out of these family physicians, roughly 34% have been retained in public sector specialist posts, 12% in public sector medical officer posts and 15% have joined the private sector. Others have emigrated (10%), left medicine altogether (11%) or taken up managerial or academic posts (10%).^2^

On the one hand, this appears something of a success story, and yet on the other hand, there is still a need for advocacy with both the public and private sectors. In the public sector, we must ensure that national human resources for health policy understands the roles and contribution of family physicians. There has been confusion over their specialist status and relationship to disciplines such as internal medicine.^3^ At the provincial level, there is variability in the commitment of sub-national government to the deployment of family physicians. This is observed particularly in our current austerity conditions and diminishing health budgets.^4^ In the private sector, there are issues with the recognition of family physicians as specialists, which has implications for both remuneration and their scope of practice.^5^ Even at the local level, there may be a need to advocate for and clarify the roles of family physicians in the health services with managers and colleagues.^6^

In this short report, we reflect on some of the lessons learnt from decades of advocacy. The World Organization of Family Doctors (WONCA) recently hosted a webinar on how to advocate with government. The presenter, Prof. Michael Kidd, outlined seven key principles for successful advocacy (Box 1).^7^ In this report, we reflect on the South African experience in terms of these seven principles.

BOX 1 Seven principles for advocacy with government.

1. Understand the issue
2. Identify the right audience
3. Build relationships
4. Use evidence and data
5. Craft a clear message
6. Engage the public
7. Use the media

*Source*: Kidd M. How to be an effective advocate with your government [homepage on the Internet]. 2024 [cited 2024 Sept 29]. Available from: https://youtu.be/z26JiQpR9sc?si=XSew4AwOs_l8TUjo

## Understand the issue

In 1994, the transition to a new democratic South Africa placed primary health care at the heart of emerging policy for a health system to serve all South Africans.^8^ The new government had a strong commitment to establishing a unified district health system with primary health care at its foundation. This shift in policy created an opportunity to advocate for the discipline of family medicine.

Successful advocacy, however, required close attention to the policy landscape and the priorities of the new government. Arguments based on European or North American models of family medicine were out of sync with the challenges facing the government – a large population without access to primary care, a health system fragmented along racial lines, a rapidly emerging human immunodeficiency virus (HIV) epidemic and insufficient and unaffordable numbers of primary care doctors.^8^ The government opted for a primary care system that was nurse-led.

The discipline of family medicine had been led and conceptualised by private practitioners. With the rapid shift in policy, medical schools started to create departments of family medicine and to expose medical students to primary care. New leadership emerged in the public sector, but needed to re-conceptualise the role of family physicians in the public health system. There was intense debate to reach consensus on the appropriate names, roles and contribution of family physicians.^9,10,11^

Successful advocacy required the discipline of family medicine to reinvent its view of itself, understand the issues facing the new government and articulate how these two emergent realities could embrace each other. In the Western Cape, for example, it was the skills gap in district hospitals and the need for a competent generalist in this setting that started to open the policy door to family medicine.^12,13^

Understanding the issue, therefore, means being an expert on your own discipline, understanding the policy landscape and seeing how your discipline can contribute to the policy priorities. As policy and priorities shift with time, this is an ongoing process. Now, 30 years later, family physicians are often invited to serve on governmental expert working groups to help re-design the policy on district health services.

## Identify the right audience

Over the years, advocacy for family medicine has become a core function of the South African Academy of Family Physicians (SAAFP).^14^ Engaging with government has required the leadership to make sense of the various statutory bodies and government departments. For example, the standing committee on health in Parliament that provides oversight, the minister and deputy minister of health, the public servants with different responsibilities in the national department of health (e.g. human resources for health, primary health care, district health system, national health insurance) and provincial heads of health. The deputy minister of health may be more available than the minister and willing to dialogue.

Identifying the right audience in the private sector has been even more complex with bodies such as the Council for Medical Schemes and a plethora of medical aid companies and private health providers. In fact, the SAAFP eventually contracted a consultancy who understood the audience better to assist with advocacy.^15^ At the same time, we established a private sector forum to listen to the needs of family physicians and create a clear constituency.

Identifying the right audience, therefore, means unpacking the different bodies, structures and organograms to identify the key people that you should engage with.

## Build relationships

Having identified the right audience, it is important to build and maintain relationships over time. This may also require some consistency in the leadership of the SAAFP. An important lesson was the need to shift from promoting the discipline of family medicine per se, to articulating how family physicians could contribute to the health system and the needs of communities.

Inviting key role players to speak at our national conference and engage us on policy developments was a useful strategy to building relationships. Our *South African Family Practice* journal also provided opportunities for government to communicate with primary care providers via editorials or explanations of new policy, such as national health insurance.^16^

As we developed relationships and gained trust, the government invited us to assist with reviewing and developing policy on task teams and working groups. The SAAFP has at times been critical, but always with the intention of being constructive and improving health care for the people of South Africa. It is best to avoid personal criticism of ministers or public servants.

## Use evidence and data

While policy development is not exclusively evidence-based, the need for evidence to support advocacy is essential. Evidence for the contribution of family medicine to health systems is therefore important and yet also scarce in the African context.^17^ There have been research studies in South Africa, but the ability to demonstrate impact has been limited by the small numbers of family physicians and their relatively recent introduction to the health system.^18^ There is little big data to enable correlational analysis such as that done by Barbara Starfield in the USA.^19^ Qualitative studies and studies examining the impact of family physicians at facility-level have been useful.^20,21^ We have also made good use of short reports to allow family physicians to tell their stories, share innovations and publish evidence that is not part of formal research.^22^ Politicians and decision makers are sometimes swayed by stories that illustrate the impact of family physicians on patients, communities or facilities.

## Craft a clear message

It is important for the discipline to speak with one voice and have ‘a clear, concise and compelling message’.^7^ The SAAFP has twice published national position papers to articulate the contribution of family physicians to the health system.^23,24^ Writing such a position paper requires building consensus and buy-in among the leadership of the discipline. The publication then provides a useful resource to share with government and can support various modes of communication such as meetings, video, podcasts or presentations.^25^ It also ensures that all advocates speak to one message. Our position papers have started by understanding the context and policy landscape, explaining how family physicians can assist, and then ending with a clear ask of what we would like government to do.

Again, the message should focus on the contribution to the health system and needs of people and not the needs of family physicians. Early on, in our advocacy journey, we compared the number of family physicians with other specialist disciplines in our conversation with government and received the feedback that we were just ‘empire building’. Government officials wanted to hear about the value to the health system and not how we compared to other disciplines.

## Engage the public

Government and other stakeholders may be persuaded to adopt new policy by civil society or campaigns led by patient or consumer groups. A good example in South Africa is the treatment action campaign that was instrumental in changing government policy and pharmaceutical profit making regarding antiretroviral treatment for HIV.^26^

Family medicine and family physicians are, however, largely unknown by the public. People do not understand the differences between general practitioners, medical officers and family physicians. The SAAFP needs to strategise and take more opportunities to communicate with the public.

## Use the media

Closely related to the previous principle is the use of media. The SAAFP has made use of opinion editorials in the general media,^27^ and publications such as the *Conversation Africa* to explain the role and contribution of family physicians.^28^ Much more could be done with regard to radio and TV to get our message out. The SAAFP must also take the opportunity to comment on current affairs and medical news. We may need to go for media training and engage media consultants to assist with this.

The *South African Family Practice* journal is the official mouthpiece of the SAAFP and makes use of editorials, opinion pieces and ‘From the President’s Desk’ to speak to the discipline. However, this is largely a conversation with ourselves, and more should be made of scientific publications such as the *South African Health Review*,^29^ *Journal of the Colleges of Medicine*^30^ and *South African Journal of Sciences* that reach a broader readership.^31^

## Conclusion

Over the last few years, the SAAFP has made progress with advocacy for family medicine and family physicians. We have developed a better understanding of the issues and policy landscape, identified key stakeholders and built relationships. We have crafted a clear position statement, which is supported by evidence and data. Much more can be done to engage the public and make use of media in explaining the role of family physicians and contribution to healthcare and health. More attention could also be given to preparing family physicians as leaders and advocates.^32^

The authors are all members of the leadership of the South African Academy of Family Physicians.
